# Datasets for lot sizing and scheduling problems in the fruit-based beverage production process

**DOI:** 10.1016/j.dib.2021.106810

**Published:** 2021-01-29

**Authors:** Juan Piñeros, Alyne Toscano, Deisemara Ferreira, Reinaldo Morabito

**Affiliations:** a Department of Production Engineering, Federal University of São Carlos, Sorocaba-SP, Brazil; b Department of Production Engineering, Federal University of Triângulo Mineiro, Uberaba-MG, Brazil; c Department of Physics, Chemistry and Mathematics, Federal University of São Carlos, Sorocaba-SP, Brazil; d Department of Production Engineering, Federal University of São Carlos, São Carlos-SP, Brazil

**Keywords:** Fruit-based beverage, Lot sizing and scheduling problems, Temporal cleaning, Pasteurized juice, Sequence dependent setups, Production planning

## Abstract

The datasets presented here were partially used in “Formulation and MIP-heuristics for the lot sizing and scheduling problem with temporal cleanings” (Toscano, A.,  Ferreira, D., Morabito, R., Computers & Chemical Engineering) [1], in “A decomposition heuristic to solve the two-stage lot sizing and scheduling problem with temporal cleaning” (Toscano, A., Ferreira, D., Morabito, R., Flexible Services and Manufacturing Journal) [2], and in “A heuristic approach to optimize the production scheduling of fruit-based beverages” (Toscano et al., Gestão & Produção, 2020) [3]. In fruit-based production processes, there are two production stages: preparation tanks and production lines. This production process has some process-specific characteristics, such as temporal cleanings and synchrony between the two production stages, which make optimized production planning and scheduling even more difficult. Thus, some papers in the literature have proposed different methods to solve this problem. To the best of our knowledge, there are no standard datasets used by researchers in the literature to verify the accuracy and performance of proposed methods or to be a benchmark for other researchers considering this problem. The authors have been using small data sets that do not satisfactorily represent different scenarios of production. Since the demand in the beverage sector is seasonal, a wide range of scenarios enables us to evaluate the effectiveness of the proposed methods in the scientific literature in solving real scenarios of the problem. The datasets presented here include data based on real data collected from five beverage companies. We presented four datasets that are specifically constructed assuming a scenario of restricted capacity and balanced costs.

## Specifications Table

**Table:** 

Subject	Management Science and Operations Research.
Specific subject area	Production Planning, Lot Sizing and Scheduling, Two-Stage Production, Temporal Cleaning, Fruit-based Beverage.
Type of data	Tables; Images; Charts; Graphs; Figures.
How data were acquired	The data were acquired through field research, observation, documents provided by companies, as well as computer generators using different probability distributions.
Data format	Raw; Analyzed; Filtered.
Parameters for data collection	The data were collected through guided tours to fruit-based beverage companies. During these visits, unstructured interviews were conducted with decision makers and employees.
Description of data collection	Initial data were obtained from field research conducted in five companies from the fruit-based beverage industry in Brazil. One of them is a multinational company. The data were collected observing their production processes using electronic spreadsheets provided by some of these companies and through interviews conducted with production managers and employees from production lines, as well as production planning and control departments. From these initial data, other instances were generated by computer generators using different probability distributions. These distributions and their respective parameters were defined according to the initially collected data. Some parameters were still adjusted aiming to have various realistic scenarios.
Data source location	City/Town/Region: São Paulo State. Minas Gerais State. Country: Brazil
Data accessibility	Repository name: Mendeley data Data identification number: 10.17632/j2 × 3 gbskfw.1 Direct URL to data: https://data.mendeley.com/datasets/j2x3gbskfw/1
Related research article	Toscano, A., Ferreira, D., Morabito, R., Formulation and MIP-heuristics for the lot sizing and scheduling problem with temporal cleanings, Computers & Chemical Engineering. Available online (2020). Doi: 10.1016/j.compchemeng.2020.107038. Toscano, A., Ferreira, D., Morabito, R., A decomposition heuristic to solve the two-stage lot sizing and scheduling problem with temporal cleaning, Flexible Services and Manufacturing Journal. 31 (2019) 142–173. Doi: 10.1007/s10696-017-9303-9. Toscano, A. , Ferreira, D., Morabito, R., Trassi, M. V. C. A heuristic approach to optimize the production scheduling of fruit-based beverages. Gestão & Produção, 27(4), e4869, 2020. https://doi.org/10.1590/0104-530x4869-20

## Value of the Data

• These datasets are useful because they are based on real data. Thus, they illustrate a more practical perspective of the problem and its complexities. Moreover, due to their variation in costs and capacity parameters, they represent different scenarios of real companies. It is well known that there is a lack of this kind of data in the scientific literature. On the other hand, research based on more realistic data has been increasingly demanded.
• This data set consists of a collection of instances for the classic problem of lot sizing and scheduling problems with sequence-dependent setup times/costs and changeover times satisfying the triangular inequality. In addition to these characteristics, there are some additional parameters such as temporal cleaning, which characterize the production processes with the pasteurization step. Therefore, studies related to the fruit-based beverage industry or other food industries with similar production process features, for example, milk production processes, can benefit from this collected data. Besides, the parameters can be used to generate instances based on real data for other lot sizing and scheduling problem classes.
• Part of the datasets was used in research concerning a fruit-based beverage industry. Therefore, the datasets provide instances for future research comparing the results and establishing a new benchmark for the problem.

## 1 Data Description

Obtaining real instances to validate methods proposed in the scientific literature still poses a challenge [4]. However, according to [5] there is a trend and a research opportunity in lot sizing and scheduling applied to real problems. Therefore, describing real data in detail is an important contribution to future research.

The dataset presented in this article describes the main parameters of a fruit-based beverage production process, which are important and should be considered when carrying out production planning and scheduling. The fruit-based production process consists of two main stages. In Stage I, raw material is mixed with water in preparatory tanks. The beverage produced in Stage I is pasteurized and filled in Stage II, called Line, thus generating the final items. These stages are dependent and must be synchronized in production planning and scheduling. Each tank in Stage I is dedicated to a production line in Stage II.

In order to change over items, cleaning is required. The times and costs for this cleaning can be sequence-dependent or independent. In these production processes, there are mandatory cleanings, also called temporal cleanings, which are necessary when the time spent from completing the last cleaning reaches a permitted maximum time without cleaning. In the beverage production process, the changeover times respect the validity of the triangular inequality, and for the fruit-based production process this characteristic is also true. Therefore, production planning and scheduling consist of deciding the quantity of each item that must be produced in each tank/line in each period and the production sequence of these items to meet the demand, minimizing backorder and inventory items, as well as the times and costs spent on temporary cleaning and cleaning by item changeovers. For more details about these production processes, see [1], [2], [3]. To create the dataset of instances, field research was conducted in five companies from the fruit-based beverage industry in Brazil, one of which is a worldwide brand.

We present a total of 92 instances divided into 4 groups (G1, G2, G3 and G4) of 23 instances each. These instances consist of real and generated data based on real data.

Although the 23 instances of the G1 group vary in size and the data are based on real information, some parameter values (such as costs and capacity) do not vary to represent some important scenarios. For example, scenarios with more restricted capacity, or scenarios where inventory and backorder costs are more balanced than changeover costs. To compose a set of more representative instances, we then generated 3 more groups of instances, G2, G3 and G4. The G2 group instances consist of the G1 instances with a reduction in time capacity by 10%. The G3 group instances were created reducing inventory and backorder costs of the G1 instances. The G4 group instances were created with modifications made in groups G1 and G2. Thus, an instance is always named by the group to which it belongs, followed by the position that it occupies in the group. For example, instance G1–15 is the fifteenth instance of Group 1.

All parameters that comprise an instance are described in Table 1. All instances are presented in electronic spreadsheets in the repository https://data.mendeley.com/datasets/j2x3gbskfw/1. More details about each group are described in the next section.

**Table 1:** Parameters of the instances.

Parameter	Description
|J|,|M|,|T|	Total number of items, tanks/lines and time periods, respectively.
J	Set of items, from 1 to |J|.
M	Set of tanks/lines, from 1 to |M|. Each line has a dedicated tank.
T	Set of periods, from 1 to |T|.
$I_{j0}^{+}\;\left(I_{j0}^{-}\right)$	Initial inventory (backorder) level for item j∈J (in units).
$tc^{I}\left(tc^{II}\right)$	Time spent to perform a temporal cleaning in a tank (in a line) (in minutes).
$te_{max}^{I}\left(te_{max}^{II}\right)$	Maximum time elapsed from the last temporal cleaning in a tank (in a line) (in minutes).
$d_{jt}$	Demand for item j∈J period t∈T (in units).
$\rho_{j}$	Quantity of beverage in one unit of item j∈J (in liters).
$cap_{mt}$	Available capacity time of tank/line m∈M in period t∈T (in minutes).
$b_{ij}^{I}\left(b_{ij}^{II}\right)$	Changeover times from item ito item j in stage I (in stage II) (in minutes).
$ub_{j\;}\left(lb_{j}\right)$	Maximum (minimum) production quantities for the lot sizes of item j∈J in the tanks (in liters).
pt	Beverage production time of one lot in a tank considered independent from the lot size (in minutes).
$s_{m}$	Time spent for line m∈M bottling one liter of beverage (in minutes).
$h_{j}^{+}\left(h_{j}^{-}\right)$	Non-negative inventory (backorder) cost for one unit of item j∈J.
$c_{ij}$	Cost of process changeover from item i to item j in the production process.
ct	Temporal cleaning cost.
$O_{mt}$	Ordered set of available lots (batches of the same item) for production in tank/line m∈M in period t∈T.
$Q_{mt}^{I}\left(Q_{mt}^{II}\right)$	Ordered set of available temporal cleanings for tank (line) m∈M in period t∈T.
$|O_{mt}|$	Maximum number of lots of the same item that can be produced in tank/line m∈M in period t∈T.
$|Q_{mt}^{I}|\left(|Q_{mt}^{II}|\right)$	Maximum number of temporal cleanings for tank (line) m∈M in period t∈T.

## 2 Experimental Design, Materials and Methods

In the data collection process, several visits were made to 5 fruit-based beverage companies from 2015 to 2019. The field research consisted of several guided tours. During these visits, a production manager from each factory described the entire process of producing fruit-based beverages. In other unstructured interviews, data were provided via some electronic spreadsheets and via information provided by the production planner. This material was then organized to generate the instances described in this paper.

The instances of group G1 are detailed in Section 2.1. The other instances (of groups G2, G3 and G4) are derived from the G1 instances and are explained in Section 2.2. These G1 group instances were used by [1].

### 2.1 G1 group of instances

The G1 group consists of the first instances generated for the problem. For each instance, some parameters were real data and others were generated based on real data. In Table 2, for each parameter, the type of data (real or generated) for each instance is specified, from 1 to 23.

**Table 2:** Classification of parameters in real data or based on real data for G1 instances.

Param.	*Instance Number*																						
	*1*	*2*	*3*	*4*	*5*	*6*	*7*	*8*	*9*	*10*	*11*	*12*	*13*	*14*	*15*	*16*	*17*	*18*	*19*	*20*	*21*	*22*	*23*
J	**✓**	**✓**	**✓**	**✓**	**✓**	**✓**	**✓**	**✓**	**✓**	**✓**	**✓**	**✓**	**✓**	**✓**	**✓**	**✓**	**✓**	**✓**	**✓**	**✓**	**✓**	**✓**	**✓**
*M*	**✓**	**✓**	**✓**	**✓**	**✓**	**✓**	**✓**	**✓**	**✓**	**✓**	**✓**	**✓**	**✓**	**✓**	**✓**	**✓**	**✓**	**✓**	**✓**	**✓**	**✓**	**✓**	**✓**
T	**✓**	**✓**	**✓**	**✓**	**✓**	**✓**	**✓**	**✓**	**✓**	**✓**	**✓**	**✓**	**✓**	**✓**	**✓**	**✓**	**✓**	**✓**	**✓**	**✓**	**✓**	**✓**	**✓**
$I_{j0}^{+}$	**✓**	**✓**	**✓**	**✓**	**✓**	**✓**	**✓**	**✓**	**✓**	**✓**	**✓**	**✓**	**✓**	**✓**	**✓**	**✓**	**✓**	**✓**	**✓**	**✓**	**✓**	**✓**	**✓**
$I_{j0}^{-}$	**✓**	**✓**	**✓**	**✓**	**✓**	**✓**	**✓**	**✓**	**✓**	**✓**	**✓**	**✓**	**✓**	**✓**	**✓**	**✓**	**✓**	**✓**	**✓**	**✓**	**✓**	**✓**	**✓**
$tc^{I}$	**✓**	**✓**	**✓**	**✓**	**✓**	**✓**	**✓**	**✓**	**✓**	**✓**	**✓**	**✓**	**✓**	**✓**	**✓**	**✓**	**✓**	**✓**	**✓**	**✓**	**✓**	**✓**	**✓**
$tc^{II}$	**✓**	**✓**	**✓**	**✓**	**✓**	**✓**	**✓**	**✓**	**✓**	**✓**	**✓**	**✓**	**✓**	**✓**	**✓**	**✓**	**✓**	**✓**	**✓**	**✓**	**✓**	**✓**	**✓**
$te_{max}^{I}$	**✓**	**✓**	**✓**	**✓**	**✓**	**✓**	**✓**	**✓**	**✓**	**✓**	**✓**	**✓**	**✓**	**✓**	**✓**	**✓**	**✓**	**✓**	**✓**	**✓**	**✓**	**✓**	**✓**
$te_{max}^{II}$	**✓**	**✓**	**✓**	**✓**	**✓**	**✓**	**✓**	**✓**	**✓**	**✓**	**✓**	**✓**	**✓**	**✓**	**✓**	**✓**	**✓**	**✓**	**✓**	**✓**	**✓**	**✓**	**✓**
$d_{jt}$	**•**	**•**	**•**	**•**	**•**	**•**	**✓**	**✓**	**•**	**•**	**•**	**✓**	**•**	**•**	**•**	**•**	**•**	**•**	**•**	**•**	**•**	**•**	**•**
$\rho_{j}$	**•**	**•**	**•**	**•**	**•**	**•**	**✓**	**✓**	**•**	**•**	**•**	**✓**	**•**	**•**	**•**	**•**	**•**	**•**	**•**	**•**	**•**	**•**	**•**
$cap_{mt}$	**•**	**•**	**•**	**•**	**•**	**•**	**✓**	**✓**	**•**	**•**	**•**	**✓**	**•**	**•**	**•**	**•**	**•**	**•**	**•**	**•**	**•**	**•**	**•**
$b_{ij}^{I}$	**•**	**•**	**•**	**•**	**•**	**•**	**✓**	**✓**	**•**	**•**	**•**	**✓**	**•**	**•**	**•**	**•**	**•**	**•**	**•**	**•**	**•**	**•**	**•**
$b_{ij}^{II}$	**•**	**•**	**•**	**•**	**•**	**•**	**✓**	**✓**	**•**	**•**	**•**	**✓**	**•**	**•**	**•**	**•**	**•**	**•**	**•**	**•**	**•**	**•**	**•**
$lb_{j}$	**•**	**•**	**•**	**•**	**•**	**•**	**✓**	**✓**	**•**	**•**	**•**	**✓**	**•**	**•**	**•**	**•**	**•**	**•**	**•**	**•**	**•**	**•**	**•**
$ub_{j\;}$	**•**	**•**	**•**	**•**	**•**	**•**	**✓**	**✓**	**•**	**•**	**•**	**✓**	**•**	**•**	**•**	**•**	**•**	**•**	**•**	**•**	**•**	**•**	**•**
pt	**•**	**•**	**•**	**•**	**•**	**•**	**✓**	**✓**	**•**	**•**	**•**	**✓**	**•**	**•**	**•**	**•**	**•**	**•**	**•**	**•**	**•**	**•**	**•**
$s_{m}$	**•**	**•**	**•**	**•**	**•**	**•**	**✓**	**✓**	**•**	**•**	**•**	**✓**	**•**	**•**	**•**	**•**	**•**	**•**	**•**	**•**	**•**	**•**	**•**
$h_{j}^{+}$	**•**	**•**	**•**	**•**	**•**	**•**	**•**	**•**	**•**	**•**	**•**	**•**	**•**	**•**	**•**	**•**	**•**	**•**	**•**	**•**	**•**	**•**	**•**
$h_{j}^{-}$	**•**	**•**	**•**	**•**	**•**	**•**	**•**	**•**	**•**	**•**	**•**	**•**	**•**	**•**	**•**	**•**	**•**	**•**	**•**	**•**	**•**	**•**	**•**
$c_{ij}$	**•**	**•**	**•**	**•**	**•**	**•**	**•**	**•**	**•**	**•**	**•**	**•**	**•**	**•**	**•**	**•**	**•**	**•**	**•**	**•**	**•**	**•**	**•**
ct	**•**	**•**	**•**	**•**	**•**	**•**	**•**	**•**	**•**	**•**	**•**	**•**	**•**	**•**	**•**	**•**	**•**	**•**	**•**	**•**	**•**	**•**	**•**
$O_{mt}$	**•**	**•**	**•**	**•**	**•**	**•**	**•**	**•**	**•**	**•**	**•**	**•**	**•**	**•**	**•**	**•**	**•**	**•**	**•**	**•**	**•**	**•**	**•**
$Q_{mt}^{I}$	**•**	**•**	**•**	**•**	**•**	**•**	**•**	**•**	**•**	**•**	**•**	**•**	**•**	**•**	**•**	**•**	**•**	**•**	**•**	**•**	**•**	**•**	**•**
$Q_{mt}^{II}$	**•**	**•**	**•**	**•**	**•**	**•**	**•**	**•**	**•**	**•**	**•**	**•**	**•**	**•**	**•**	**•**	**•**	**•**	**•**	**•**	**•**	**•**	**•**

**✓** Real data

**•** Data generated based on real data.

#### 2.1.1 Real data

In the companies visited, an item is considered a six-pack with 6 bottles of a certain beverage. The total number of items |J| varied in the set {3,4,5,6,10,15,20}, the total number of preparation tanks/lines |M| varied in the set {2,4,6}, and the total number of periods |T| varied in the set {2,4,5,6}. These data are real for all instances (Table 2). The size of each instance was determined by the number of items, tanks/lines and periods. They are shown in Table 3.

**Table 3:** Number of items, tanks/lines and periods for G1 instances.

	*Instance Number*																						
Parameter	*1*	*2*	*3*	*4*	*5*	*6*	*7*	*8*	*9*	*10*	*11*	*12*	*13*	*14*	*15*	*16*	*17*	*18*	*19*	*20*	*21*	*22*	*23*
|J|	3	3	3	3	3	4	5	5	5	5	5	5	6	10	10	10	10	15	15	15	20	20	20
|M|	2	2	2	2	2	2	2	2	2	2	2	2	2	2	4	4	4	6	6	6	6	6	6
|T|	2	2	2	2	2	4	4	4	4	4	4	5	4	4	4	4	4	4	4	4	6	6	6

Among the 5 companies visited, 3 of them can be considered small and medium sizes, mainly due to the number of items produced and machines available for production, and the volume of production. They are represented in Table 3 by instances 1 to 6. The other 2 companies are large and are represented by instances 7 to 23.

We also observed in the field research that the initial inventory and backorder levels for all items were always zero, that is, $I_{j0}^{+}=0$ and $I_{j0}^{-}=0$, for all instances. The time spent on performing a temporal cleaning was 50 min for stage I and 300 min for stage II in all companies visited. Thus, these values are real for all instances, $tc^{I}=50$ and $tc^{II}=300$. The same occurs for the maximum time elapsed since the last temporal cleaning in the line and tank. These values were identical for all companies and therefore, they are real data for all instances, with $te_{max}^{I}=1,445$ and $te_{max}^{II}=2,885\;\text{min}$, respectively.

It can be observed in Table 2 that most parameters of instances G1–7, G1–8, and G1–12 are real data. These three instances were collected from a worldwide brand of a fruit-based beverage whose plant is located in São Paulo State, Brazil. For these instances, we collected the demands of five items (orange, passion fruit, grape, strawberry and pineapple). Instances G1–7, G1–8 and G1–12 correspond to one month each, non-consecutive, divided into weeks (periods). Notice that the month of instance G1–12 has 5 weeks. For all items from these instances, the quantity of beverage, in liters, to produce one unit of any item *j*∈J is $\rho_{j}=2.4$. For these instances, the available capacity time for each tank/line m∈M in each period t∈T is 8550 min.

For instances G1–7, G1–8 and G1–12, the changeover times from item i to item j in both stages ($b_{ij}^{I}$ and $b_{ij}^{II}$) are fixed and sequence-independent. This fact was observed only in this company. For the other companies, these times are sequence-dependent and are presented in the next section. These instances are the same real instances used by [2] and [3]. We also collected the lower and upper bounds for the lot sizes,$\;lb_{j}$ and $ub_{j}$, respectively, defined by the physical capacities of the tanks. The time spent to produce the beverage in Stage I (pt) and the bottling time of one liter in line m∈M ($s_{m}$) were also collected. For more details of these parameter values, please see the Supplementary Material.

#### 2.1.2 Estimated data based on interviews

Not all parameters were provided by the companies. Some of them were generated based on real information collected in the companies visited. For example, some parameters were randomly generated using probability distributions, such as the normal and uniform continuous distributions. These parameters and the respective probability distributions are shown in Table 4. The variation intervals for each parameter were defined according to the information and data obtained in the five companies visited.

**Table 4:** Parameters generated using probability distributions.

Parameter	Distribution
$\rho_{j}$	U[0.9;12]
$d_{\alpha t}$	$U[\frac{min}{\rho_{i}};\frac{max}{\rho_{i}}]\;$
$d_{\beta t}$	$U[\frac{max}{\rho_{j}};\frac{2\;\times \;max}{\rho_{j}}]$
$d_{jt}$	$U\left[0;\frac{\;\text{li}m_{\text{jt}}}{\rho_{j}}\right],\;\forall \;t\in T,\;\forall \;j\in \;J\;\setminus \;\left\{\alpha ,\beta \right\}.\;$
${b^{I}}_{ij}$	$N(30,5^{2})$
${b^{II}}_{ij}$	$N(150,30^{2})$
$ub_{j}$	U[10,000;20,000]
$lb_{j}$	$\theta ub_{j}$ and θ∼U[0.20;0.6]
pt	$N(100,10^{2})$
$s_{m}$	U[40;200]

For parameters $d_{jt}$, $\rho_{j}$, $ub_{j}$, $lb_{j}$ and $s_{m}$, the collected samples were small. Due to the experience and suggestion of the production planner interviewed, we used the uniform distribution to generate these parameters, which proved to be a reasonable option for this purpose. The limits of the generation interval for each one of these parameters were defined based on the collected data and on the decision-makers´ knowledge.

In some cases, from more significant samples for the changeover times $\left(b_{ij}^{I},\;b_{ij}^{II}\right)$ and the production times of a batch ($p_{t}$), we computed descriptive statistics, the mean and standard deviation, and visually verified that the data approached a symmetric distribution. For this reason and based on the recommendation of the interviewed decision-maker, we consider it reasonable to use the normal distribution to generate these data. It should be mentioned that nonparametric fit tests were not performed.

As mentioned, an item is considered a six-pack with 6 bottles of a certain beverage, therefore the quantity of beverage in one unit of item j ($\rho_{j}$), in liters, can vary between 0.9 and 12 liters.

In order to ensure that the instances generated show the need to schedule temporal cleaning, the demands are generated to guarantee that at least two items have this characteristic.

The demand generation aims to ensure that there will be at least one temporal cleaning in the period. To reach that, initially two items called α and β were randomly chosen in the set of items J to have their demands generated as shown in Table 4, whereby $min={}_{\forall m\;\in M}^{min}\left\{\left[s_{m}\times te_{max}^{II}\right]\right\}$ and $max={}_{\forall m\;\in M}^{max}\left\{\left[s_{m}\times te_{max}^{II}\right]\right\}$. The value $s_{m}\times te_{max}^{II}$ is an estimate of the possible beverage quantity so that their demands could be filled without needing to perform a temporal cleaning in stage II. The min and max parameters are, respectively, the smallest and biggest numbers of liters that can be filled in $te_{max}^{II}$ minutes among all lines m∈M. These values are used to generate the demand of items and it is guaranteed that there is at least one item whose production requires a temporal cleaning.

A uniform continuous distribution varying in [0, $\text{li}m_{\text{jt}}$] ∀t∈T,∀j∈J∖{α,β} was used to generate the other item demands. The $\text{li}m_{\text{jt}}$ is calculated by:

(1) $$\text{li}m_{\text{jt}}=\frac{cap\_disp_{t}}{\left|J\right|-\;2},\;\forall \;t\in T,\;\forall \;j\in \;J\;\setminus \left\{\alpha ,\beta \right\},$$

where:

(2) $$cap\_disp_{t}=\;\left(\sum_{m\;\in M}8,550\;\times \;s_{m}\right)-\;\sum_{j\;\in \;\left\{\alpha ,\beta \right\}}\rho_{j}\times \;d_{jt}.$$

Parameter $cap\_disp_{t}$ is an estimate for the available capacity after the production of items α and β. It was calculated based on the real production capacity of the period (i.e., 8550 min).

The changeover of different items in the tanks consists of a cleaning for which the time spent (${b^{I}}_{ij}$) is, on average, 30 min, depending on the production sequence. Thus, it is considered that this value varies according to a normal distribution with mean 30 and standard deviation 5. Similarly, the changeover in the filling machines (${b^{II}}_{ij})$ is a cleaning. The time taken for a changeover of items i and j, in the lines, follows a normal distribution with mean 150 and standard deviation 30. These values were generated ensuring the validity of the triangular inequality.

In the production processes of the companies, we found tanks with different capacities in liters, from 10,000 liters to 20,000 liters. The maximum production quantities ($ub_{j}$) were defined in this range. The minimum lot size quantity ($lb_{j}$) depends on the minimum quantity of raw material that is used to produce it, which varies for different flavors and ranges from 20% to 60% of the maximum lot size.

In the different factories visited, the beverage production time (pt) of one lot in a tank was around 100 min with variations of 20 min, on average. Therefore, this parameter was generated following a normal distribution with a standard deviation of 10 min. The filling machine speed ($s_{m})$ found in the visited companies had a speed between 40 and 200 liters per minute. The available capacity, in minutes, for each preparation tank/line m∈M and each period t∈T, was generated based on papers [6] and [7]. This capacity value is:

(3) $$cap_{mt}=cap_{mt}^{\prime }+\;\frac{ca{p^{\prime }}_{mt}}{te_{max}^{II}}\times tc^{II},$$

where $cap_{mt}^{\prime }$ is an estimate for the time spent on a possible temporal cleaning that takes place throughout the period. The $cap_{mt}^{\prime }$ value can be calculated by:

(4) $$cap_{mt}^{\prime }=\left(\sum_{j\;\in J}\frac{d_{jt}\;\rho_{j}}{\sum_{m\;\in M}s_{m}}\right)+\left|J\right|\;\times \;\begin{array}{c} max\\ \forall i,j\in J \end{array}\;\left\{b_{\text{ij}}^{\text{II}}\right\}+tc^{II}$$

In the companies visited, there was common ground for production costs. All companies wished to obtain a production plan that optimizes production costs: backorders, inventories, changeovers and temporal cleanings. Nevertheless, the unit costs of each of them are hard to be precisely estimated by the companies in practice. Thus, the companies use a system of priorities for each minimized term of the objective function when determining a production plan. They considered that the economic loss caused by backlogging was much higher than the inventory cost, which in turn was much higher than the opportunity costs caused by changeover and temporal cleaning. Therefore, the costs were generated in order to properly represent these priorities, in unit penalties. Table 5 presents the values of these parameters.

**Table 5:** Penalties of the production process.

Parameter	InstancesG1–7, G1–8, G1–12.	Instances: G1–1 to G1–6;G1–9 to G1–11; G1–13 to G1–23.
$h_{j}^{+}$	10	U[10;20]
$h_{j}^{-}$	100	$10\;\times {h_{j}}^{+}$
$c_{ij}$	1	$10\;\times \;(\frac{{b^{I}}_{ij}+\;{b^{II}}_{ij}}{\begin{array}{c} max\;\\ \forall i,j\in J\setminus i_{0} \end{array}\;\left\{{b^{I}}_{ij}+\;{b^{II}}_{ij}\right\}\;})$
ct	1	1

**Table 6:** Production lots for the production plan of instance G1–7.

Flavor	t=1		t=2		t=3		t=4	
	Number of lots (Liters per lot)		Number of lots (Liters per lot)		Number of lots (Liters per lot)		Number of lots (Liters per lot)	
	m=1	m=2	m=1	m=2	m=1	m=2	m=1	m=2
Orange					2 (5000.00)			
					1 (4483.33)			
					1 (3999.99)			
					1 (3866.66)			
					19 (2666.66)			
					1 (2573.33)			
					1 (1250.00)			
Passion					1 (5000.00)	3 (5000.00)		
Fruit					1 (2666.66)	1 (4333.33)		
					2 (2500.00)	4 (2500.00)		
Grape	2 (4167.08)	1 (4167.08)	8 (4167.08)	5 (4167.08)			12 (2666.66)	11 (2166.66)
	2 (4000.00)	1 (3250.00)	8 (2083.33)	1 (4091.66)			14 (4167.08)	1 (4167.08)
	2 (3866.66)	1 (3091.25)	4 (2666.66)	20 (2166.66)			4 (2083.33)	1 (2083.33)
	1 (3214.16)	20 (2166.66)	1 (2326.25)	8 (2083.33)			1 (3866.66)	1 (3141.66)
	34 (2666.66)	4 (2083.33)	1 (3866.66)				1 (2131.25)	
	11 (2083.33)						1 (2864.16)	
Strawberry	3 (5000.00)	5 (5000.00)	4 (5000.00)	4 (5000.00)	1 (4000.00)		19 (2666.66)	13 (2166.66)
	1 (4533.33)	1 (4283.33)	1 (4866.66)	1 (4231.66)	1 (3400.00)		2 (5000.00)	14 (5000.00)
	1 (3351.66)	1 (3975.00)	1 (2666.66)	1 (1666.66)	1 (3866.66)		1 (4000.00)	1 (2641.66)
	6 (2666.66)	1 (3250.00)	5 (1666.66)		23 (2666.66)		1 (4066.66)	1 (2475.00)
	7 (1666.66)	10 (2166.66)			1 (2573.33)		1 (1790.00)	
		1 (2106.66)			1 (1666.66)		1 (1666.66)	
		1 (1666.66)					1 (3866.66)	
Pineapple			2 (5000.00)	6 (5000.00)				
			1 (4533.33)	1 (3153.33)				
			2 (2666.66)	4 (2500.00)				
			7 (2500.00)

**Table 7:** Parameters that are identical and different in G1, G2, G3 and G4 groups of instances.

	Group			
Parameter	G1	G2	G3	G4
J	❍	❍	❍	❍
*M*	❍	❍	❍	❍
T	❍	❍	❍	❍
$I_{j0}^{+}$	❍	❍	❍	❍
$I_{j0}^{-}$	❍	❍	❍	❍
$tc^{I}$	❍	❍	❍	❍
$tc^{II}$	❍	❍	❍	❍
$te_{max}^{I}$	❍	❍	❍	❍
$te_{max}^{II}$	❍	❍	❍	❍
$d_{jt}$	❍	❍	❍	❍
$\rho_{j}$	❍	❍	❍	❍
$cap_{mt}$	❍	◆	❍	◆
$b_{ij}^{I}$	❍	❍	❍	❍
$b_{ij}^{II}$	❍	❍	❍	❍
$lb_{j}$	❍	❍	❍	❍
$ub_{j\;}$	❍	❍	❍	❍
pt	❍	❍	❍	❍
$s_{m}$	❍	❍	❍	❍
$h_{j}^{+}$	❍	❍	◆	◆
$h_{j}^{-}$	❍	❍	◆	◆
$c_{ij}$	❍	❍	❍	❍
ct	❍	❍	❍	❍
$O_{mt}$	❍	❍	❍	❍
$Q_{mt}^{I}$	❍	❍	❍	❍
$Q_{mt}^{II}$	❍	❍	❍	❍
N	❍	❍	❍	❍

❍ - Original ◆ - Changed.

**Fig. 1:**
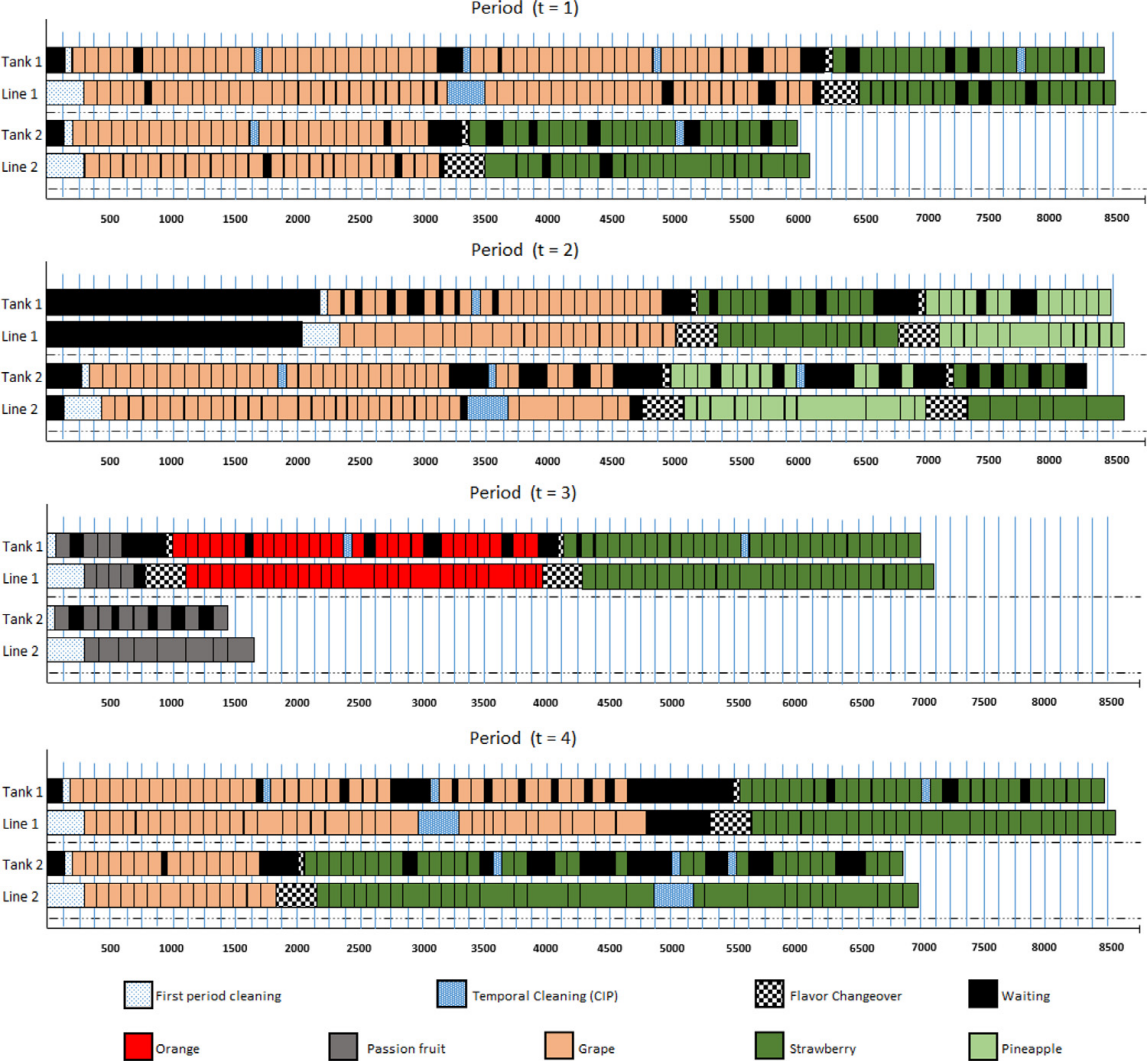
Gantt chart for the production plan of instance G1–7.

**Table 8:** Description of G1, G3, G3 and G4 groups of instances.

Group	Description	Modification
G1	Original created instances	–
G2	Instances of G1 group with 10% reduction in capacity	0.9 $\times cap_{mt}$
G3	Instances of G1 group with adjusted backorder and inventory costs	${h_{j}}^{+}∼\;U\left[0.5;\;2\right]$ ${h_{j}}^{-}=\left(20\;\times {h_{j}}^{+}\right)+Max\left[c_{ij}\right]$
G4	Instances of G1 group with 10% reduction in capacity and adjusted backorder and inventory costs	0.9 $\times cap_{mt}$ ${h_{j}}^{+}∼\;U\left[0.5;\;2\right]$ ${h_{j}}^{-}=\left(20\;\times {h_{j}}^{+}\right)+Max\left[c_{ij}\right]$

For the real instances G1–7, G1–8 and G1–12, the values were fixed as shown in Table 5. The unit penalties were determined trying to fairly represent the company's main goal, which is to supply the demand of all customers with no backlogging. The smallest penalties were for changeover and temporal cleanings. Thus, we set the backlogging cost to 100 per unit, the inventory cost to 10 per unit, the temporal cleaning cost to 1 per unit and the changeover to 1 per unit. These values were validated by the company managers. For the other 20 instances, each minimized term followed the same priorities. However, the values for these penalties were based on the other collected data in the other companies, as presented in Table 5.

As the tanks have a limited capacity, a maximum number of lots (batches) was estimated that can be prepared in a period. An estimate for the number of available lots (batches) of the same item for production in tank/line m∈M in period t∈T was calculated by equation:

(5) $$\left|O_{mt}\right|=\frac{\left[cap_{mt}-\left(\frac{cap_{mt}}{te_{max}^{II}}+1\right)\times tc^{II}\right]\times s_{m}}{0.75\;\times \;max_{j\in J}\left\{ub_{j}\right\}}$$

In (5), the maximum number of lots is provided by the division of the line *m* period *t* capacity to produce an item discounting the time spent on cleaning (denominator of the equation) by the nominal capacity of the tank (numerator of the equation).

It was assumed that only item j is produced throughout the period and that there is an estimate of time spent on possible temporal cleanings due to the large production of item j in the period, $\left(\left(\frac{cap_{mt}}{te_{max}^{II}}+1\right)\times tc^{II}\right)$. We discounted the estimate time spent on temporal cleanings from the available capacity of line *m* in period *t*, $cap_{mt}$ $\left(cap_{mt}-\left(\frac{cap_{mt}}{te_{max}^{II}}+1\right)\times tc^{II}\right)$. To calculate this final period capacity in liters per minutes, it was multiplied by the filling speed of line $\left(s_{m}\right)$. In addition, to obtain an estimate for the maximum number of lots that can be produced in line m in the period t ($|O_{mt}|$), this estimate of the effective production capacity of line m in period t, in liters (numerator of the fraction (5), is divided by 75% of the largest maximum lot ($ub_{j}).$ The value of 75% was obtained from the visited companies. In the companies, the nominal capacity is 75% of the real tank capacity. Finally, set $O_{mt}$ ranges from 1 to $|O_{mt}|$ for each item j∈J.

The number of possible temporal cleanings that can occur in Stages I and II were $|Q_{mt}^{I}|$ and |$Q_{mt}^{II}|$, respectively, calculated by Eqs. (6) and (7):

(6) $$\left|Q_{mt}^{I}\right|=\frac{cap_{mt}}{te_{max}^{I}},$$

(7) $$\left|Q_{mt}^{II}\right|=\frac{cap_{mt}}{te_{max}^{II}}\;.$$

Therefore, set $Q_{mt}^{I}$ ranges from 1 to $|Q_{mt}^{I}|$ and set $Q_{mt}^{II}$ ranges from 1 to $|Q_{mt}^{II}|$.

All parameters described above for each instance are available in spreadsheets in the Supplementary Material.

#### 2.2 Generation of G2, G3 and G4 groups of instances

In order to evaluate the instances generated for the G1 group, we created a manual production plan for instance G1–7. In this G1–7 solution, the demands of all items are met in all periods, that is, there are no backorders and inventories in this plan. Table 6 presents the number of lots and the corresponding liters for each item that must be produced in each machine and period. Notice, for example, that in machine m=1 of period t=1, 52 lots of the Grape item and 18 lots of the Strawberry item are planned to be produced.

The Gantt chart for this production plan is shown in Fig. 1.

In Fig. 1, the horizontal line is the production timeline and each rectangle indicates the time spent on producing a lot, or waiting, or cleaning, etc. It can be observed that there is available capacity in almost all machines and periods, and the demand is met without backorders and inventories. For example, in line m=2 of period t=3, the production finishes in less than 2000 min, still leaving more than 6550 available minutes of the production capacity. This fact suggests that we can decrease the available capacity $\left(cap_{mt}\right)$ and obtain instances with more restricted capacity. Therefore, we generated instances from group G2 reducing the value of $cap_{mt}$, for all m∈M and tϵT, of each G1 instances by 10%, i.e., G2 instances are based on G1 instances, except for $cap_{mt}$.

In G1 group instances, the inventory and backorder costs are much more penalized than the changeover cost, as these are unit costs. However, for different companies, it is more important to meet the demand required for the period, either with inventory or production during this period than not meeting the customer demand in the required period and losing credibility with that customer. To represent these situations, the G3 group instances were created reducing inventory and backorder costs of the G1 instances with$\;{h_{j}}^{+}∼\;U\left[0.5;\;2\right]$ and ${h_{j}}^{-}=\left(20\;\times {h_{j}}^{+}\right)+Max\left[c_{ij}\right]$, for all j∈J, respectively. To obtain even more realistic scenarios, the G4 instance group was also created with modifications made in groups G1 and G2, i.e., they have the value of $cap_{mt}$ reduced by 10% and ${h_{j}}^{+}∼\;U\left[0.5;\;2\right]$ and ${h_{j}}^{-}=\left(20\;\times {h_{j}}^{+}\right)+Max\left[c_{ij}\right]$. Table 7 presents the parameters that are identical to G1 and the parameters that have been changed for each group.

A summary of the characteristics of the four groups of instances G1, G2, G3 and G4 is presented in Table 8.

## Declaration of Competing Interest

The authors declare that they have no known competing financial interests or personal relationships that could have appeared to influence the work reported in this paper.
